# Use of Therapeutic Plasma Exchange during Extracorporeal Life Support in Critically Ill Cardiac Children with Thrombocytopenia-Associated Multi-Organ Failure

**DOI:** 10.3389/fped.2017.00254

**Published:** 2017-12-01

**Authors:** Mei Chong, Alejandro J. Lopez-Magallon, Lucas Saenz, Mahesh S. Sharma, Andrew D. Althouse, Victor O. Morell, Ricardo Munoz

**Affiliations:** ^1^Department of Critical Care Medicine, Division of Cardiac Intensive Care, Children’s Hospital of Pittsburgh of UPMC, Pittsburgh, PA, United States; ^2^Department of Pediatric Heart Center, Beijing Anzhen Hospital, Capital Medical University, Beijing Institute of Heart, Lung, and Blood Vessel Diseases, Beijing, China; ^3^Department of Cardiothoracic Surgery, Children’s Hospital of Pittsburgh of UPMC, Pittsburgh, PA, United States; ^4^UPMC Heart and Vascular Institute, Pittsburgh, PA, United States

**Keywords:** plasma exchange, children, congenital heart disease, extracorporeal membrane oxygenation, multiple organ failure, TAMOF

## Abstract

**Background:**

Thrombocytopenia-associated multi-organ failure (TAMOF) in children is a well-described factor for increased hospital mortality. Low cardiac output syndrome (LCOS) and the effects of cardiopulmonary bypass may manifest with several adverse physiologic and immunologic effects, with varying degrees of thrombocytopenia and multi-organ dysfunction, sometimes very similar to TAMOF. LCOS is a common occurrence in children with critical heart disease, presenting in as much as 23.8% of infants postoperative of congenital heart surgery. Therapeutic plasma exchange (TPE) has been offered as a promising therapy for TAMOF; however, the therapeutic implications of this modality in children with critical heart disease and a clinical diagnosis of TAMOF are unknown.

**Objectives:**

We describe our institutional experience with TPE as an adjuvant rescue therapy for children with critical heart disease and a clinical diagnosis of TAMOF, while supported by extracorporeal membrane oxygenation (ECMO).

**Methods:**

Single-center retrospective analysis of children with critical heart disease admitted to the CICU and supported by ECMO, undergoing TPE for a clinical diagnosis of TAMOF between January 2006 and June 2015.

**Results:**

Forty-one patients were included for analysis. Median age and weight of patients was 0.6 years (range 0.0–17.2) and 8.5 kg (range 1.5–80.0). TPE was initiated at a median of 1 day (0–13) after initiation of ECMO. Modified organ failure index (MOFI) and platelet count improved after TPE start (*p* < 0.001). Patients with early TPE initiation after ECMO cannulation (<1 day) showed more improvement in MOFI and platelet counts than patients with late TPE initiation (*p* < 0.001 for each). Overall survival to hospital discharge was 53.7%. The within-groups hospital survival was 73.3% for patients with heart failure, 34.8% for patients with congenital heart disease, and 100% for those with other cardiac disease (*p* = 0.016).

**Conclusion:**

In children with critical cardiac disease and clinical diagnosis of TAMOF necessitating ECMO for hemodynamic support, concurrent TPE may be associated with an improvement in organ failure and platelet count, particularly when started early. Further studies are warranted to establish the most effective use of TPE and its effect on survival in this population.

## Introduction

Multiple organ dysfunction syndrome (MODS) is a well-described and common condition in critically ill children (1), including patients with infectious and non-infectious systemic inflammatory response syndrome and those with heart disease. It has been reported in as much as 84.6% of patients at the time of their intensive care unit admission (2); hospital mortality remains high and is progressively worse depending on the number of failing organs (3–5). In addition, a distinct subset of very sick patients presents with clinical manifestations of Thrombocytopenia-associated multi-organ failure (TAMOF) (6). In this condition, patients develop a secondary thrombotic microangiopathy associated with a decreased activity of a protease (disintegrin and metalloproteinase with thrombospondin motifs 13, ADAMTS-13), leading to increased circulating ultra-large von Willebrand factor units (vWF), platelet overconsumption and organ failure secondary to vWF-rich microvascular thromboses. These patients are at exceptionally high risk of death and have been previously reported in the pediatric population (7–9).

Given the dire prognosis of TAMOF, additional therapeutic resources aiming to improve survival include extracorporeal membrane oxygenation (ECMO) and therapeutic plasma exchange (TPE). The use of TPE has been described as adjuvant therapy for critically ill patients presenting with TAMOF in small randomized controlled trials and case series (7–9), and it is hypothesized that its benefit may be related to removal of cytotoxins, dysregulated cytokines, and restoration of deficient or depleted humoral products including ADAMTS-13.

Recently, Kawai et al. reported a single-center case series of patients with a clinical diagnosis of TAMOF due to sepsis that underwent combined ECMO and TPE therapy. This strategy portends the theoretical advantage of providing sustained cardiopulmonary support while enhancing organ recovery. Interestingly, these patients had a higher than expected survival (9).

In children with critical medical or perioperative heart disease, low cardiac output syndrome (LCOS) is a common occurrence, presenting in as much as 23.8% of infants postoperative of congenital heart disease (10). It has been described as a condition associated with intrinsic heart disease, myocardial ischemia from aortic cross-clamp and the effects of cardiopulmonary bypass (CPB), resulting in a decreased systemic perfusion state as well as several adverse physiologic and immunologic effects (11, 12). When left untreated, a varying degree of extra-cardiac organ failure may complicate the clinical picture resulting in cardiac arrest, the need for ECMO, a prolonged respiratory failure, and an increased mortality (13).

The use of ECMO to provide mechanical cardiopulmonary support for children with critical cardiopulmonary failure is a well-established rescue therapy for children with critical medical or postoperative heart disease (14). The overall reported survival rate to hospital discharge for pediatric cardiac patients supported by ECMO ranges from 38 to 73% with good functional results (15, 16). However, a lower survival rate has been associated in patients supported with this therapy whenever used in the presence of MODS. The international Extracorporeal Life Support Organization report has published survival rates of 19% for neonatal cardiac patients that underwent dialysis during ECMO, and as low as 17% for those with concomitant DIC (17). Some of these patients may share physiologic, immune response, and hematologic features with non-cardiac patients with TAMOF and it is unknown whether they could benefit with a similar approach. In fact, a decreased ADAMTS-13 activity has been reported in cardiac patients after heart surgery with CPB (18).

We have previously reported our institutional outcomes with the use of ECMO in children with critical heart disease, including an intermediate survival rate of 66% with most survivors having normal or mild neurodevelopmental deficit at follow-up (16). In this study, the use of TPE was identified as an independent variable significantly associated with an increased risk for mortality. However, no further analysis was done for this subgroup of patients. During the last few years, we developed a systematic approach for children with critical heart disease with LCOS and a clinical diagnosis of TAMOF while supported by ECMO, including the use of TPE. To our knowledge, its use in this population has not been previously characterized and we describe here our institutional experience as we wanted to inform ourselves in anticipation of a potential prospective randomized controlled trial.

## Materials and Methods

The study was performed in the cardiac intensive care unit of Children’s Hospital of Pittsburgh of UPMC, a tertiary academic center with an active heart–lung transplantation and mechanical circulatory support program. A detailed description of our program as well as short and intermediate outcomes in our patients supported by cardiac ECMO has been previously published (16).

### Simultaneous ECMO and TPE Procedures

Therapeutic plasma exchange procedures were done always by the transfusion/apheresis team. Routine laboratories collected before and after the procedure included: hemoglobin, platelet (PLT), international normalized ratio, pH, PaO2, Heparin anti-factor Xa activity, activated partial thromboplastin time. Most of our patients under ECMO usually received heparin drips to keep an adequate systemic anticoagulation; therefore, we did not use further anticoagulation for the procedure. However, in those cases where patient’s systemic anticoagulation was sub-therapeutic (i.e., fresh postoperative patients with active bleeding), we used citrate within the TPE circuit, in these cases iCa++ levels were continuously monitored and titrated as needed with a calcium chloride or gluconate drip depending on availability. We used heparin-coated circuits in all the cases.

We used two apheresis systems: COBE^®^ Spectra (software version 4.7 or 5.1, Terumo BCT, Lakewood, CO, USA) and, more recently, the Spectra Optia^®^ (software version 6.1, Terumo BCT, Lakewood, CO, USA). The blood inflow to the plasmapheresis device is connected to a positive port immediately next to the outflow of the centrifugal pump and the outflow from the plasmapheresis system is connected to a positive port downstream from the centrifugal pump (upstream to the oxygenator) in a parallel fashion.

For pediatric cases, RBC priming of the circuit was utilized, no divert prime and no rinse back was done. Most cases had 1.0–1.5 plasma exchange volumes and most of the procedures had taken place in approximately 2–2 1/2 h. All cases received plasma as fluid replacement. Fluid balance was set at 100% for both systems in all the procedures. The ECMO circuit volume was not used for calculations.

### Patient Selection

After receiving approval from University of Pittsburgh Institutional Review Board, Pittsburgh, PA, USA, IRB # PRO12070044, we conducted a descriptive, retrospective review of our ECMO database and associated medical records of all the pediatric surgical or medical cardiac patients (<18 years) admitted to our CICU and receiving TPE for clinical diagnosis of TAMOF while on ECMO from January 2006 to June 2015. Typically, a decision to offer TPE was made at the discretion of the attending cardiac intensivist in consensus with the cardiac surgeon. In addition, we retrospectively calculated a previously described modified organ failure index (MOFI) for all the patients, adding 1 point separately for cardiovascular and pulmonary failure (9). We did not include neurologic failure as a part of the organ failure calculations since all patients were sedated. Therefore, the maximum score a patient could have accumulated was 5. Patients >18 years, those with a platelet count of >100,000 or a MOFI of <2 during the first day of TPE (as independently assessed by one of our coinvestigators), were excluded from analysis (7, 9).

Collected data included demographic information, cardiac diagnosis and surgery, risk adjustment in congenital heart surgery (RACHS-1), hospital length of stay, indication for ECMO, indication and number of TPE sessions received, performed surgical procedures, and associated laboratory values. For each patient, we registered daily MOFI as well as the worse daily values of platelet count (PLT), lactate, creatinine, bilirubin, alanine transaminase (ALT), aspartate transaminase (AST), prothrombin time (PT), and ADAMTS-13. Primary outcomes were the change in MOFI and platelet count after receiving TPE, with survival to discharge from the hospital as secondary outcome.

Patients were divided into three major categories based on indication for ECMO: E-CPR (cannulation during cardiopulmonary resuscitation refractory to conventional resuscitation), OR-ECMO (failure to wean from CPB in the operating room), and LCOS-ECMO (refractory LCOS) (3). In addition, patients were grouped into the following subgroups: (a) heart failure (including primary myocardial disease and history of OHT before admission); (b) congenital heart disease (including single-ventricle and two-ventricle lesions); (c) other cardiac diseases (including primary pulmonary hypertension, arrhythmia, subacute bacterial endocarditis, etc.).

### Statistical Methods

If a patient had multiple hospital admissions and multiple ECMO runs, only the first hospitalization and ECMO run was included in the analysis. For descriptive statistics of the study population, continuous variables are summarized as median (range); categorical variables are summarized as frequency (percentage). Changes in MOFI and platelet count are shown over time (indexed to the day of TPE initiation) as mean ± SE; linear mixed-effects models were used to test for changes over time in MOFI and platelet count, with time as a fixed effect and subject as a random effect, to properly account for the repeated measurements within subjects. Interactions with time were used to test for differences between groups (TPE start 0–1 days after ECMO vs TPE start >1 day after ECMO). Survival to hospital discharge is shown as frequency (percentage) overall and within selected subgroups. All statistical analyses were performed using SAS version 9.4 (SAS Institute, Cary, NC, USA).

## Results

### Study Population

During the study period from January 1, 2006 to June 30, 2015, there were a total of 2,853 surgical admissions to the cardiac intensive care unit, with 136 patients requiring ECMO support. We identified 51 cardiac patients receiving TPE while on ECMO during our study period. Ten patients were excluded for different reasons (4 > 18 years, 6 with no TAMOF criteria during the initial day of TPE). In the end, 41 patients were included for analysis: 14 (34.1%) females and 27 (65.9%) males. When divided by cardiac diagnostic group, 15 (36.6%) patients were included in the heart failure and 23 (56.1%) in the congenital heart disease groups, respectively. Most of the patients had two-ventricle physiology (30, 72.3%) (Table 1). There were 29 (70.7%) surgical patients and 12 (29.2%) medical patients. As related with surgical complexity, 15 (51.7%) patients had a RACHS-1 score ≥3. Indications for ECMO support included E-CPR in 17 (41.4%) patients, LCOS-ECMO in 17 (41.4%), OR- ECMO in 6 (14.6%), and 1 (2.4%) with ECMO cannulation for respiratory failure. TPE was started <1 day after ECMO cannulation in 25 (56.8%) patients, and >1 day after cannulation in 19 (43.2%). The overall survival to hospital discharge was 53.7% (Table 2).

**Table 1:** Baseline characteristics of study patients (on ECMO day 1).

Variable	Total population
# Patients	41
Age (Years)	0.6 (0.0–17.2)
**Gender**	
Female	14 (34.1%)
Male	27 (65.9%)
**# Ventricles**	
Univentricular	11 (26.8%)
Biventricular	30 (72.3%)
**Diagnostic Category**	
Heart Failure	15 (36.6%)
Congenital Heart Disease	23 (56.1%)
Other	3 (7.3%)
Height (cm)	70.0 (40.5–180.0)
Weight (kg)	8.5 (1.5–80.0)
Creatinine	0.8 (0.2–2.0)
Bilirubin	1.9 (0.3–12.0)
ALT	52.5 (4.0–3521)
AST	131 (13.0–4200)
Lactate	5.7 (1.5–19.0)
Platelet count	70.0 (8.0–267)
PT	23.0 (8.6–88.3)
MOFI	3.0 (2.0–5.0)
SvO_2_	70.0 (20.0–94.0)

*Continuous variables presented as median (range). Categorical variables presented as *n* (%)*.

*ALT, alanine transaminase; AST, aspartate transaminase; PT, prothrombin time; MOFI, modified organ failure index; SvO_2_, mixed venous oxygen saturation; ECMO, extracorporeal membrane oxygenation*.

**Table 2:** ECMO + TPE + hospital stay characteristics.

Variable	Total population
# Patients	41
Surgical	29 (70.7%)
**ECMO type**	
OR-ECMO	6 (14.6%)
LCOS	17 (41.5%)
E-CPR	17 (41.5%)
Respiratory failure	1 (2.4%)
ECMO run (hours)	120 (24.0–600)
ECMO run (days)	5.0 (1.0–25.0)
ECMO to TPE time (days)	1.0 (0.0–10.0)
**TPE initiation**	
0–1 days after ECMO	24 (58.5%)
>1 day after ECMO	17 (41.5%)
**# TPE sessions**	
1	6 (14.6%)
2	11 (26.8%)
3	7 (17.1%)
4	7 (17.1%)
5	4 (9.8%)
≥6	6 (14.6%)
Length of CICU stay (days)	38.0 (2.0–257)
CICU survival	23 (56.1%)
Length of hospital stay (days)	54 (2–278)
Hospital survival	22 (53.7%)

*Continuous variables presented as median (range); Categorical variables presented as *n* (%)*.

*ECMO, extracorporeal membrane oxygenation; OR-ECMO, cannulation for failure to separate from cardiopulmonary bypass in the operating room; LCOS, low cardiac output syndrome; ECPR, Cannulation during refractory cardiopulmonary resuscitation; CICU, cardiac intensive care unit. TPE, therapeutic plasma exchange*.

### MOFI and Laboratory Variables before and after TPE

**Figure 1:**
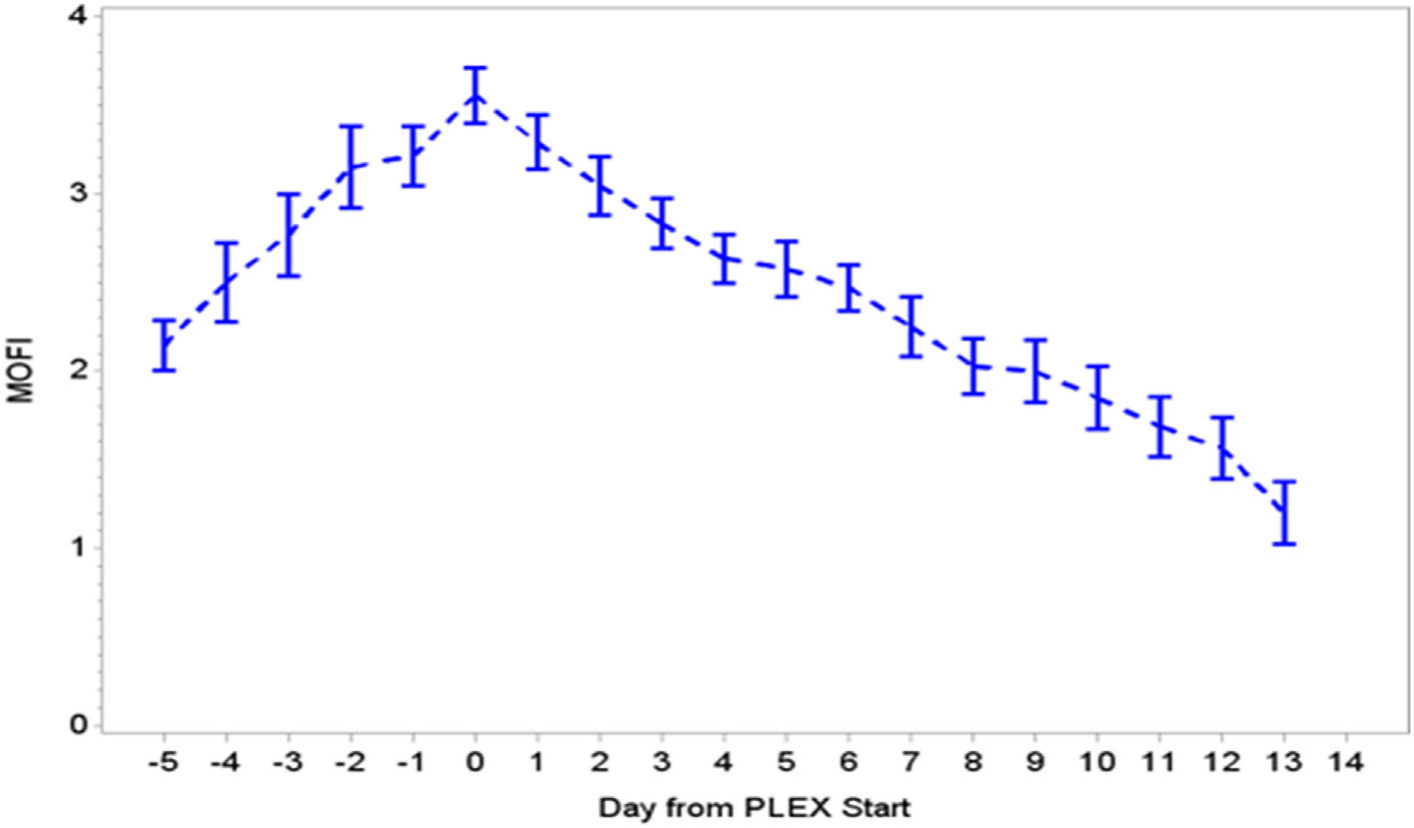
Change in MOFI during PLEX (*p* < 0.001 for change over time). MOFI, modified organ failure index; PLEX, therapeutic plasma exchange.

**Figure 2:**
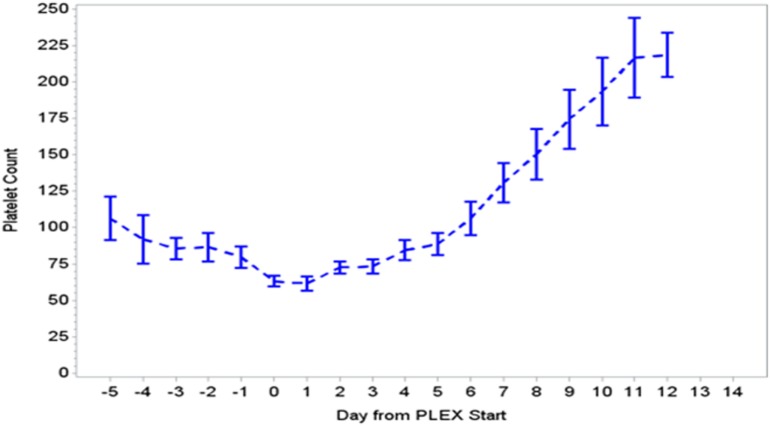
Change in platelet count during PLEX (*p* < 0.001 for change over time). PLEX, therapeutic plasma exchange.

**Figure 3:**
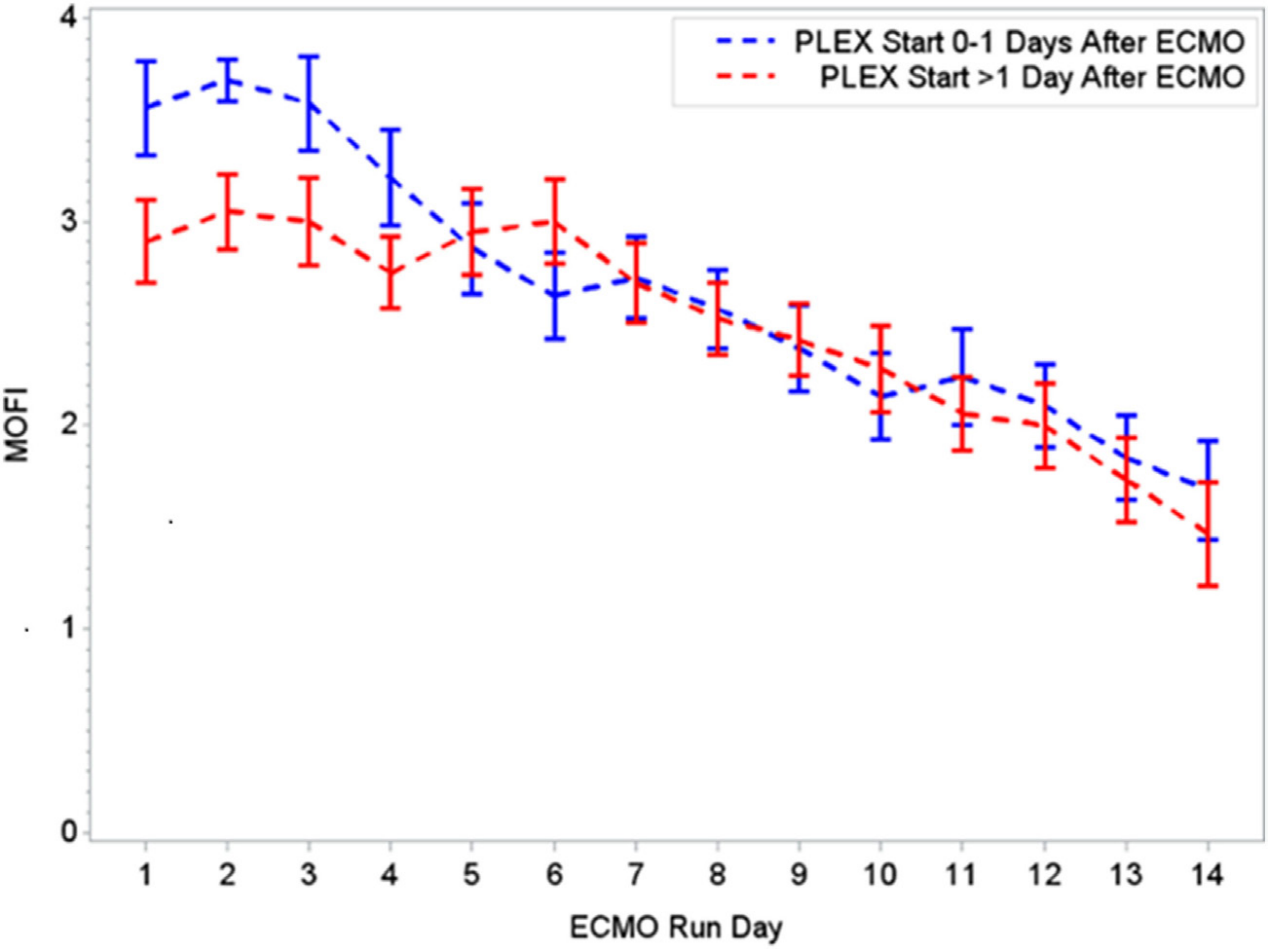
Changes in MOFI per early (0–1 days) or late (>1 day) PLEX start after ECMO. MOFI, modified organ failure index. ECMO, extracorporeal membrane oxygenation; PLEX, therapeutic plasma exchange.

**Figure 4:**
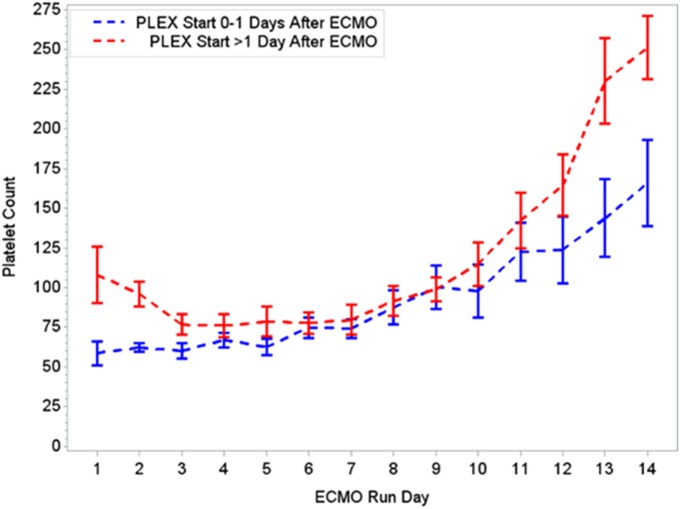
Changes in platelet count per early (0–1 days) or late (>1 day) PLEX start after ECMO. ECMO, extracorporeal membrane oxygenation; PLEX, therapeutic plasma exchange.

During the first ECMO day, the median value for MOFI was 3.0 (range 2.0–5.0) and for platelet count 70,000/mm^3^ (range 8,000–267,000). After TPE start, there were significant changes over time with a decrease in MOFI and an increase in platelet count (*p* < 0.001 each, Figures 1 and 2). Other laboratory values showing significant changes over time after ECMO cannulation included ALT, AST, lactate, and PT (*p* < 0.001 for each). We performed a subgroup analysis dividing patients per early (<1 day after ECMO cannulation) or late (>1 day after cannulation) TPE start. Patients with early TPE start showed a significantly faster decrease in daily MOFI as well as an increase in daily platelet count compared with patients with late TPE start (*p* < 0.001 each, Figures 3 and 4).

### Survival to Discharge within Selected Patient Subgroups

The within-groups hospital survival per diagnostic category showed a significantly higher survival in the heart failure group (11/15, 73.3%) as compared with the congenital heart disease one (8/23, 34.8%, *p* = 0.007). When analyzed by cardiovascular physiology group, two-ventricle physiology patients showed a significantly higher survival (19/30, 63.3%) when compared with univentricular patients (3/11, 27.3%) (*p* = 0.04). Finally, analysis by ECMO type showed a significantly higher hospital survival in ECPR and LCOS-ECMO patients (11/17, 64.7% and 11/17, 64.7%, respectively) as compared with OR-ECMO (0/6 survivors, 0%) (*p* = 0.021). There was no significant difference in hospital survival for age, gender, or surgical vs non-surgical status (Table 3).

**Table 3:** Survival to discharge within selected patient subgroups.

Variable	# Patients	Survival to discharge	*p*-Value
# Patients	41	22	
**Age (Years)**			
0–1	21	9 (42.9%)	0.423
1–5	9	5 (55.6%)	
5–10	6	4 (66.7%)	
>10	5	4 (80.0%)	
**Gender**			
Female	14	7 (50.0%)	0.735
Male	27	15 (55.6%)	
**# Ventricles**			
Univentricular	11	3 (27.3%)	0.040
Biventricular	30	19 (63.3%)	
**Diagnostic category**			
Heart failure	15	11 (73.3%)	0.016
Congenital heart disease	23	8 (34.8%)	
Other	3	3 (100%)	
**Surgical status**			0.077
Non-surgical	12	9 (75.0%)	
Surgical	29	13 (44.8%)	
**ECMO type**			0.021
OR-ECMO	6	0 (0.0%)	
LCOS	17	11 (64.7%)	
ECPR	17	11 (64.7%)	
Respiratory failure	1	0 (0.0%)	
**TPE initiation**			0.576
0–1 days after ECMO	24	12 (50.0%)	
>1 day after ECMO	17	10 (58.8%)	

*Continuous variables presented as median (range); Categorical variables presented as *n* (%)*.

*ECMO, extracorporeal membrane oxygenation; OR-ECMO, cannulation for failure to separate from cardiopulmonary bypass in the operating room; LCOS, low cardiac output syndrome; ECPR, cannulation during refractory cardiopulmonary resuscitation; CICU, cardiac intensive care unit; TPE, therapeutic plasma exchange*.

## Discussion

In children with heart disease and critical cardiopulmonary failure, ECMO support remains the most important rescue therapy. However, LCOS is commonly present as a complication and in a distinct subset of patients their clinical presentation is consistent with TAMOF. They may benefit from additional therapy with TPE and these two therapies are well tolerated together (9).

In our group of patients, we observed a significant improvement in MOFI and platelet count over time after initiation of TPE therapy as revealed by our time-effect analysis, although further study is needed to determine whether this is truly additive over the benefits of ECMO alone. Furthermore, the rapid improvement in MOFI and platelet count in our patients with early TPE therapy resembles previous reports suggesting that early initiation of TPE may be more beneficial. Kawai et al. described a trend toward a decreased ECMO run duration when TPE was initiated early, nonetheless the association was limited due to their small cohort of patients (9). Also, Akca et al. reported an increased mortality in adult patients with thrombocytopenia when low platelet count persisted at day 14 post ICU admission (19). Alternatively, patients with higher MOFI scores (sicker patients) at the time of ECMO cannulation may have warranted an earlier intervention. These findings have potential therapeutic implications, but the descriptive nature of our study does not allow us to establish whether TPE is truly beneficial or a bystander to other interventions like ECMO or ultrafiltration (a less expensive potential alternative for some patients), among others.

A few additional observations are also worth mentioning. Overall survival for this cohort of patients was higher than what would be expected given their serious clinical condition. This could be explained based on a difference in the distribution of several features between our patient group and those in previous reports on overall survival for children with cardiac disease supported by ECMO and specifically related to surgical status, univentricular vs two-ventricle physiology, diagnostic group category, or reason for ECMO cannulation. Indeed, hospital survival for patients with myocarditis (mostly two-ventricle physiology) is the highest among cardiac patients receiving ECMO (20). On the opposite side, patients with congenital heart disease have exhibited a comparatively low survival (15, 17). Whether these patients would further benefit with TPE, and which group would benefit the most, are important questions that need an answer. Particularly, our patient cohort included an important proportion of them with two-ventricle physiology (72.3%) as well as those categorized in the heart failure group (36.6%), and both subgroups presented a significantly higher hospital survival rate when compared with the ones with univentricular physiology or congenital heart disease subgroups, respectively. These findings are in alignment with a previous report from our team, reporting a higher survival for patients with two-ventricle physiology compared with those with a single ventricle (16). Finally, the significantly lower hospital survival in our patients cannulated in the OR compared with previous reports from our center and others (16, 21) provides support to the concept of a higher risk for patients undergoing a surgical procedure with unmitigated multi-organ failure.

### Study Limitations

Given the retrospective nature of our study, a heterogeneous population and the lack of a control group, we are unable to discern whether these changes are truly related to TPE or other unaccounted and concurrent interventions. Also, the study period spread over 9 years and it is possible that changes in technology and advances in the design of circuit and oxygenators were contributory to the overall improved outcomes. A decision to initiate and end TPE was at the discretion of the CICU intensivist in consensus with the cardiac surgeon. Oftentimes, termination of TPE was based on the decision to decannulate the patient. With our typically short ECMO runs, this resulted in a highly irregular and shorter TPE regime. It is also possible that unaccounted differences in platelet transfusion therapy had an independent effect on platelet count trends. A systemic inflammatory response and platelet consumption are well-described occurrences during CPB and ECMO (22, 23) and it is possible that some of the findings (especially platelet count) in some of our patients were related with this phenomenon, as opposed with true TAMOF. Lastly, we did not control for ADAMTS-13 activity and it is possible that the findings in some patients were an associated effect of CPB and not TAMOF. Despite these limitations, we believe our findings justify the need for a larger, prospective randomized multicenter study with appropriate controls to identify indications, subgroups of patients that may benefit the most from the use of TPE and potential adverse sequelae.

## Conclusion

Therapeutic plasma exchange was successfully used in children with cardiac disease and a clinical diagnosis of TAMOF while supported by ECMO. The overall survival to hospital discharge was 53.7%. This therapy when used with ECMO appeared to be associated with significant organ failure recovery, and earlier initiation after ECMO cannulation was associated with a greater initial improvement in organ dysfunction. These data support the need for a larger, multicenter prospective RCT to identify overall benefit and indications, best timing of therapy start and which population subgroups are most likely to exhibit a positive response.

## Author Note

Presented at the 8th World Congress of the World Federation of Pediatric Intensive & Critical Care Societies (PICC 2016), Toronto, ON, Canada, on June 4–8, 2016.

## Author Contributions

Conception and design: MC, AL-M, and RM. Analysis and interpretation: MC, AL-M, LS, MS, AA, VM, and RM. Manuscript writing: MC, AL-M, LS, MS, and AA. Critical revision of manuscript: MC, AL-M, LS, MS, AA, VM, RM. Data collection: MC. Provision of materials, patients, or resources: MS, VM, and RM. Statistical expertise: AA. Literature search: MC, AL-M, LS, and RM.

## Conflict of Interest Statement

The authors declare that the research was conducted in the absence of any commercial or financial relationships that could be construed as a potential conflict of interest.

## Abbreviations

ACT, activated clotting time; ADAMTS-13, a disintegrin and metalloproteinase with thrombospondin motifs 13; ALT, alanine transaminase; APTT, activated partial thromboplastin time; AST, aspartate transaminase; CI, confidence interval; CPB, cardiopulmonary bypass; CPR, cardiopulmonary resuscitation; ECLS, extracorporeal life support; ECMO, extracorporeal membrane oxygenation; E-CPR, ECMO cannulation during refractory cardiopulmonary resuscitation; LCOS, low cardiac output syndrome; MODS, multiple organ dysfunction syndrome; MOFI, modified organ failure index; OHT, orthotropic heart transplant; OR, odds ratio; OR-ECMO, ECMO indication for failure to separate from cardiopulmonary bypass; TPE, therapeutic plasma exchange; PLT, platelet count; PT, prothrombin time; SvO_2_, mixed venous oxygen saturation; TAMOF, thrombocytopenia-associated multi-organ failure; VAD, ventricular assist device; vWF, von Willebrand factor.
